# Efficient Dermoscopic Lesion Segmentation via Multi-Directional State-Space Modeling and Frequency-Aware Boundary Refinement

**DOI:** 10.3390/jimaging12080339

**Published:** 2026-07-27

**Authors:** Zhengqi Liu, Lijun Xu

**Affiliations:** School of Computer Science, Hubei University, Wuhan 430062, China; 202331116020219@stu.hubu.edu.cn

**Keywords:** state-space model, multi-directional scanning, frequency-domain decoupling, boundary attention, linear-complexity segmentation, skin lesion segmentation, dermoscopy

## Abstract

State-space models segment images in linear time, but existing dermoscopic segmenters serialize the two-dimensional feature map along only one or two scan directions and operate purely in the spatial domain, which dilutes the orientation cues and fine boundary information that distinguish a pigmented lesion from surrounding skin. We address both limitations in a single linear-complexity network, Hydra-DermSeg-Net, that unifies three components not previously combined for this task: a four-directional bidirectional Hydra block that aggregates forward and backward selective scans over horizontal, vertical and two diagonal trajectories with learnable fusion weights; a differentiable discrete cosine transform (DCT) branch that decouples high- and low-frequency content so that boundary detail is processed separately from global semantics; and a learnable local contrast-enhancement front-end coupled with a clDice-supervised boundary attention gate. On a merged ISIC 2017/2018 corpus of 3994 images, the model attains a Dice coefficient of 0.9041 and an Intersection-over-Union (IoU) of 0.8386 against five baselines (U-Net, Att-UNet, VM-UNet, TransUNet and MALUNet) trained from scratch under a unified protocol, and it transfers to an unseen HAM10000 subset at 0.9347 Dice. It attains the highest Dice and IoU on each of the three data partitions examined, the highest Sensitivity, and the lowest 95-percentile Hausdorff distance under every random seed; the margin over the strongest convolutional baselines is about 0.002 in Dice and lies within the variation between training runs. An ablation at the full model capacity isolates the contribution of each component: removing the frequency branch costs 0.86 points of Dice and 0.99 points of Boundary IoU, and removing the boundary attention gate 0.66 and 2.85 points respectively. These results show that combining multi-directional state-space scanning with frequency-domain decoupling yields accurate and parameter-efficient segmentation without the quadratic cost of self-attention.

## 1. Introduction

Cutaneous melanoma is one of the most aggressive skin cancers worldwide; the latest GLOBOCAN report estimates over 330,000 new cases and approximately 60,000 deaths in 2022 alone [1,2]. The prognosis depends critically on the disease stage at first diagnosis: when localized melanoma is detected and excised early, the five-year survival rate exceeds 99%, whereas the same metric drops to below 35% once distant metastasis has occurred. Dermoscopy is the most widely used non-invasive imaging modality for the early examination of pigmented lesions, but the manual delineation of lesion borders is time-consuming, suffers from substantial inter-observer variability, and remains inherently dependent on the experience of the clinician [3,4]. An automated and reliable segmentation pipeline is therefore a prerequisite for any clinically useful computer-aided diagnosis (CAD) system, ranging from quantitative shape and border analysis to downstream malignant–benign classification.

Segmenting skin lesions in dermoscopic images, however, remains a particularly challenging task in medical image analysis. Pigmented lesions vary enormously in size, shape and pigmentation pattern, from small ephelides covering only a few hundred pixels to large irregular melanomas that occupy most of the field of view. The contrast against surrounding skin is frequently low, and borders often fade gradually into healthy tissue, leaving pixel-level decisions unstable near the margin. Acquisition adds its own difficulties: hair occlusion, specular reflections from immersion fluid, gel residues and uneven illumination all corrupt local appearance cues. Such conditions have been documented repeatedly [3,4] and continue to limit the deployment of automated segmenters in clinical workflows.

Over the past decade, deep-learning-based segmenters have been developed to address these difficulties. Convolutional encoder–decoder backbones, in particular the U-Net family [5,6,7], showed that skip connections can combine multi-scale appearance cues with high-resolution localization. Context- and boundary-aware extensions such as CE-Net [8] further enlarged the effective receptive field through dense feature pyramids. More recently, vision Transformers [9,10] have been incorporated into the U-shape, exchanging convolutional locality for global self-attention. Although these architectures have steadily pushed Accuracy on public dermoscopic benchmarks, two recurring limitations remain. (i) Stacked convolutions enlarge the receptive field only gradually, which is sub-optimal when lesions span a substantial fraction of the image. (ii) Global self-attention scales quadratically with the spatial resolution of the input, leading to a prohibitive memory footprint when high-resolution dermoscopic images are used, and consequently to the need for aggressive downsampling that sacrifices fine boundary cues.

State-space models (SSMs), in particular the Mamba family [11,12] and the bidirectional Hydra block [13], provide a third route. Mamba2 introduces a structured state-space duality (SSD) operator that models long-range dependencies in linear time and constant memory; Hydra extends Mamba2 by performing forward and backward selective scans through a quasiseparable matrix mixer, retaining the linear cost while modeling the bidirectional context required for visual recognition. Several recent works have adapted SSMs to medical image segmentation, including VM-UNet [14], AC-MambaSeg [15] and ASP-VMUNet [16] for skin lesion analysis, OCTAMamba [17] for retinal vasculature, as well as variants built on extended LSTM and Receptance Weighted Key Value formulations [18,19,20]. These studies report that SSM-based segmenters can match or exceed transformer baselines while using substantially fewer parameters and a lower computational budget.

Despite this rapid progress, several open issues remain for SSM-based dermoscopic segmentation. Existing methods predominantly rely on a single-direction scan, or at best two directions, to serialize the two-dimensional feature map. For dermoscopy this is a poor fit, because pigmented lesions present arbitrarily oriented borders and a fixed-axis serialization dilutes precisely the directional cues that separate lesion from background. A second gap concerns the frequency domain. Most recent SSM-based models operate entirely in the spatial domain, even though earlier wavelet and discrete cosine transform (DCT) studies [21,22,23] established that high- and low-frequency content carry different structural information; so far, no Mamba- or Hydra-style backbone has exploited this split in a unified, end-to-end differentiable way. Finally, illumination heterogeneity and partial occlusion (hair, gel, glare) are usually handled by fixed pre-processing—most commonly contrast-limited adaptive histogram equalization (CLAHE) with hand-tuned hyper-parameters—which is decoupled from the segmentation objective and therefore passes sub-optimal contrast on to the downstream network.

So far these issues have been tackled one at a time: multi-direction scanning, frequency-domain decoupling and learnable contrast enhancement each appear in separate models, yet no dermoscopic segmenter brings them together within a single linear-complexity backbone. We close this gap with Hydra-DermSeg-Net, a U-shaped network that combines four design choices in one framework. (1) A four-directional Hydra block extends bidirectional state-space modeling to horizontal, vertical, and two diagonal scanning paths, so that the network can follow the arbitrary orientation of lesion borders in linear complexity. (2) A parallel discrete cosine transform branch separates high- and low-frequency components, preserving fine boundary cues alongside the global semantic context. (3) A learnable local contrast-enhancement (LCE) front-end, inspired by CLAHE but fully differentiable, normalizes local contrast end-to-end in place of the conventional fixed pre-processing pipeline. (4) A boundary attention gate, coupled with a topology-aware clDice loss [24], enforces explicit edge consistency, which helps most on ambiguous lesion borders.

We evaluate Hydra-DermSeg-Net on a multi-source dermoscopic training corpus constructed by merging the ISIC 2017 [25] and ISIC 2018 [3] datasets after de-duplication, comprising 3994 unique images. Cross-domain generalization is further assessed on a held-out HAM10000 subset [4]. Under fair, from-scratch training and identical input resolution, the proposed model achieves a Dice coefficient of 0.9041 and an Intersection-over-Union (IoU) of 0.8386 on the merged test split, the highest of the six models evaluated, and it reaches 0.9347 Dice on the unseen HAM10000 subset. Ablation studies confirm that the DCT branch and the boundary attention module each contribute measurable, non-overlapping gains.

The contributions of this paper can be summarized as follows.

- We propose a four-directional bidirectional state-space block tailored for dermoscopic segmentation, in which forward and backward selective scans are aggregated across horizontal, vertical and two diagonal directions with learnable fusion weights, enabling the network to follow the arbitrary orientation of lesion contours in linear complexity.
- We design two complementary modules that explicitly address the long-standing challenges of dermoscopic imaging: a differentiable two-dimensional DCT auxiliary branch that decouples high- and low-frequency cues to enhance boundary localization and global semantic representation, and an end-to-end learnable local contrast-enhancement front-end coupled with a boundary attention gate supervised by a topology-aware clDice loss, jointly mitigating illumination heterogeneity and ambiguous lesion borders.
- We construct a multi-source ISIC 2017+2018 evaluation protocol with strict de-duplication and benchmark the model against five representative baselines spanning convolutional (U-Net, Att-UNet), transformer (TransUNet), state-space (VM-UNet) and lightweight (MALUNet) families under a unified from-scratch protocol. The proposed model attains the highest Dice, IoU and Sensitivity and the lowest Hausdorff distance among all baselines, its margin over the strongest convolutional baselines being about 0.002 in Dice and within the variation between training runs. It transfers to a held-out HAM10000 subset without fine-tuning, at roughly one quarter of the parameters of the strongest transformer baseline.

The remainder of this paper is organized as follows. Section 2 reviews related work on skin lesion segmentation, state-space models and frequency-domain learning. Section 3 details the architecture of Hydra-DermSeg-Net and its constituent modules. Section 4 describes the datasets, evaluation protocol and implementation details. Section 5 reports the quantitative and qualitative results, including ablation and cross-domain analyses. Section 6 discusses the findings, and Section 7 concludes the paper.

## 2. Related Work

### 2.1. Convolutional and Transformer-Based Skin Lesion Segmentation

Convolutional neural networks (CNNs) have dominated skin lesion segmentation since the introduction of fully convolutional networks. The original U-Net architecture [5] popularized a symmetric encoder–decoder structure with skip connections, allowing fine spatial details to be re-injected into the decoder and remains the most influential baseline in medical image segmentation. UNet++ [7] refined this design by re-engineering the skip connections into a nested, dense aggregation that mitigates the semantic gap between encoder and decoder features. Attention U-Net (Att-UNet) [6] further introduced gated attention modules on the skip pathway, suppressing irrelevant background activations and concentrating capacity on the region of interest. To enlarge the effective receptive field, CE-Net [8] combined dense atrous convolutions with a residual multi-kernel pooling block, achieving notable gains in retinal and skin lesion benchmarks. Along the same line, Liu et al. [26] trained an encoder–decoder segmentation network jointly with an auxiliary task, using the shared representation to sharpen pigmented-lesion boundaries in dermoscopic images. To meet the latency and memory budgets of edge devices, lightweight U-shaped variants such as MALUNet [27] aggressively reduce the channel widths and replace the standard convolutional stem with a stack of multi-attention modules, achieving a parameter count below 0.2 M while remaining competitive on dermoscopic benchmarks; in a similar spirit, LTPNet [28] fused three lesion-aware feature paths within a single decoder to recover lesion structures accurately at a modest computational budget. More recently, hybrid pipelines that pair a convolutional feature extractor with metaheuristic feature selection and a downstream machine learning classifier have also been explored for interpretable skin-cancer detection [29]. While these convolutional variants progressively pushed Accuracy upwards, they all share an intrinsic locality: long-range dependencies between distant pixels can only be modeled implicitly through repeated downsampling and stacking, which is sub-optimal for lesions whose boundary cues span a large portion of the image.

To overcome this locality, recent works have hybridized the U-shape with vision Transformers. TransUNet [9] replaced the deepest encoder stage with a vision Transformer, demonstrating that global self-attention can substantially improve segmentation Accuracy on multi-organ CT data and ISIC dermoscopy alike. Swin-UNet [10] subsequently constructed a fully transformer-based U-Net using shifted-window self-attention, alleviating the cost of dense attention while retaining global context modeling. These hybrids have steadily raised the state of the art on the ISIC challenges; however, they pay a steep computational price: the memory footprint of vanilla self-attention grows quadratically with the number of tokens, and even windowed variants still incur a sizable parameter overhead. As a result, transformer-based segmenters are often impractical for deployment on portable or edge devices that are increasingly being explored for tele-dermatology.

Boundary-aware modeling has emerged as a parallel line of research. XBound-Former [30] explicitly couples multi-scale boundary embeddings with a pyramid transformer, achieving state-of-the-art results on ISIC and PH^2^ benchmarks. WA-Net [22] demonstrates that wavelet-aware encoders can further refine ill-defined lesion borders, and EG-Net [20] integrates an explicit edge extraction module with an RWKV-based global modeler for melanoma segmentation. Most recently, CGQR-Net [31] introduced a contour-guided, query-based feature-fusion scheme for boundary-aware and cross-dataset-generalizable cardiac ultrasound segmentation; although it targets a different modality, it reinforces the same two principles that guide the present work—explicitly injecting contour information into the network, and assessing boundary quality under a cross-dataset domain shift. The recurrent appearance of boundary-focused designs in the recent literature indicates that pixel-wise classification loss alone is insufficient to capture the topological coherence of lesion contours, a finding that directly motivates the boundary attention gate proposed in this work.

### 2.2. State-Space Models for Medical Image Segmentation

Selective state-space models (SSMs), popularized by Mamba [11] and its successor Mamba2 [12], model long-range dependencies in linear time and have rapidly diffused into vision tasks. Mamba endows each token with a learnable selection mechanism that decides which past states should be propagated forward, while Mamba2 reformulates the recurrence as a structured state-space duality (SSD) operator that admits a highly efficient matrix-multiplication form. Building on these foundations, several U-shaped segmenters have been proposed for medical imaging. VM-UNet [14] was among the first to embed Mamba-style blocks inside a four-stage U-shape, demonstrating competitive Accuracy on multi-organ benchmarks at a fraction of the cost of comparable transformer baselines. AC-MambaSeg [15] introduced an adaptive convolutional gating mechanism into the Mamba block, while ASP-VMUNet [16] proposed an atrous shifted parallel design specifically for ISIC skin lesion analysis. Beyond skin imaging, OCTAMamba [17] adapted the Mamba block to optical coherence tomography angiography, confirming the versatility of state-space modeling across imaging modalities.

Most closely related to the present work, the Hydra block [13] extends Mamba2 with a bidirectional selective scan grounded in quasiseparable matrix mixers, allowing forward and backward sequence dynamics to be aggregated within a single linear-time operator. Parallel research has explored alternative linear-complexity sequence operators in medical segmentation, including the extended LSTM (xLSTM) backbone of VMAXL-UNet [18] and the Receptance Weighted Key Value (RWKV) formulation underlying BSBP-RWKV [19] for medical image segmentation and EG-Net [20] for melanoma analysis. Although these works collectively demonstrate the promise of linear-complexity sequence operators, they almost uniformly employ a one- or two-direction scan along the rasterized feature map. In contrast, the multi-direction Hydra block proposed in this paper aggregates four scanning trajectories with learnable fusion weights, an extension that we empirically show to be beneficial for arbitrarily oriented lesion contours.

### 2.3. Frequency-Domain and Boundary-Aware Designs

The discrete cosine transform [23] and wavelet decomposition have a long history in classical image processing, where they have been used to disentangle texture from coarse structure. Recent deep models have rediscovered these tools as a means of injecting frequency priors into convolutional or attention-based backbones. FFM-Net [21] demonstrated that a frequency-fused Mamba block improves segmentation Accuracy by enhancing low- and high-frequency components separately before fusing them into the spatial pathway. WA-Net [22] adopted a wavelet-aware encoder to selectively preserve high-frequency cues during downsampling, leading to improved boundary localization in skin lesion images. A recurring observation underlies these results: high-frequency components tend to encode object boundaries while low-frequency components carry semantic context, so separating the two benefits dense prediction. What is striking across the literature reviewed above is that the three relevant lines of work remain disconnected. Multi-direction scanning has been explored for state-space backbones, frequency decoupling has been bolted onto convolutional or single-scan Mamba models, and contrast enhancement is still treated mostly as a pre-processing step. Bringing these strands into one linear-complexity backbone is the gap that motivates the present work.

The boundary side of segmentation has been studied through both metric design and loss engineering. The Boundary IoU metric [32] explicitly evaluates Accuracy on a narrow band around the contour, complementing pixel-wise Dice/IoU that are insensitive to small but topologically critical errors. The topology-aware clDice loss [24], originally proposed for tubular structure segmentation, has been adopted in numerous downstream tasks because it penalizes disconnected components and reinforces the centerline of the predicted mask. Pre-processing has similarly evolved from fixed CLAHE [33] towards differentiable variants that can be jointly trained with the segmentation network. Hydra-DermSeg-Net draws on this body of work, combining a frequency-aware DCT branch, a learnable contrast-enhancement front-end and a clDice-supervised boundary attention gate within a single state-space backbone—an integration of all three components that we have not seen reported elsewhere for dermoscopic skin lesion segmentation.

## 3. Materials and Methods

### 3.1. Overall Architecture

**Figure 1:**
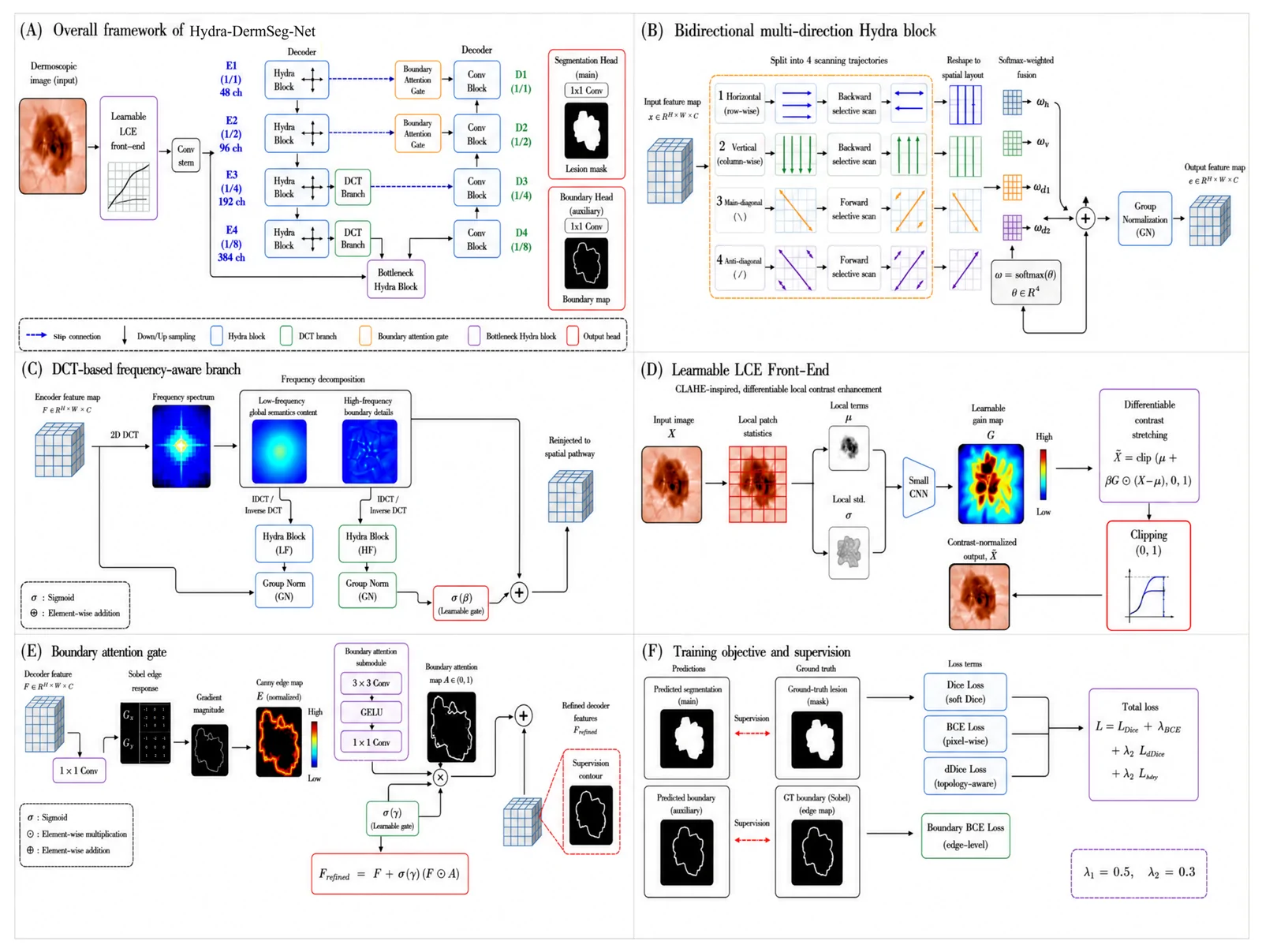
Architecture of Hydra-DermSeg-Net. (**A**) Overview of the U-shaped encoder–decoder: The input passes through the learnable local contrast-enhancement (LCE) front-end, four Hydra blocks with progressive downsampling, a DCT auxiliary branch attached to the two deepest encoder stages, and a boundary attention gate on the decoder path. (**B**) The bidirectional four-direction Hydra block, showing the horizontal, vertical and two diagonal scans and their learnable fusion. (**C**) The DCT-based frequency-aware branch and the separation of low- and high-frequency bands. (**D**) The learnable LCE front-end. (**E**) The boundary attention gate. (**F**) The training objective, combining Dice, BCE, clDice and boundary BCE terms.

Hydra-DermSeg-Net is a four-stage U-shaped encoder–decoder built on top of bidirectional state-space blocks. The complete architecture is illustrated in Figure 1. An input image $X\in R^{3\times H\times W}$ is first processed by a learnable local contrast-enhancement (LCE) front-end, yielding a contrast-normalized image $\tilde{X}$ that is fed into a small convolutional stem. The stem output is then routed through four encoder stages with channel widths $\left(C_{1},C_{2},C_{3},C_{4}\right)=\left(48,96,192,384\right)$. Each stage contains a multi-direction Hydra block that aggregates four selective-scan trajectories, and the two deepest stages additionally house a parallel DCT branch. A bottleneck Hydra block sits at the lowest resolution. The decoder mirrors the encoder via transposed convolution and skip connections. The two upper decoder stages additionally pass through a boundary attention gate, which weights the decoder features by a coarse edge map estimated from a side-output prediction. The final segmentation head produces a single-channel logit map at the input resolution, and an auxiliary boundary head supervises edge consistency.

### 3.2. Bidirectional Multi-Direction Hydra Block

Let $x\in R^{B\times L\times D}$ denote a sequence representation of a flattened feature map with L=HW tokens and *D* channels. A standard Mamba2 block [12] computes y=SSD(x), where SSD denotes the state-space duality operator. The Hydra block [13] extends this by additionally processing the reversed sequence and aggregating both:

(1) $$y_{\mathrm{Hydra}}=g\cdot \mathrm{SSD}\left(x\right)+\left(1-g\right)\cdot \mathrm{flip}\left(\mathrm{SSD}(\mathrm{flip}(x))\right),$$

where g=σ(α) is a learnable sigmoid gate. While Equation (1) captures bidirectional context along one scanning axis, a two-dimensional dermoscopic feature map admits multiple natural traversal orders. To exploit this, our multi-direction Hydra block serializes the feature map x along four trajectories using a direction-specific scan operator $S_{k}={\mathrm{Scan}}_{k}\left(x\right)$, namely horizontal row-major (k=h), vertical column-major (k=v), main diagonal ($k=d_{1}$) and anti-diagonal ($k=d_{2}$), applies the bidirectional Hydra operation of Equation (1) to each serialized sequence, and restores every output to the spatial layout via the inverse mapping ${\mathrm{Unscan}}_{k}$. The four spatial maps are then fused by a softmax-normalized weight vector $\omega \in R^{4}$:

(2) $$F_{\mathrm{multi}}=\mathrm{GN}\left(x+\sum_{k\in \{h,v,d_{1},d_{2}\}}\omega_{k}\;{\mathrm{Unscan}}_{k}\left(\mathrm{Hydra}(S_{k})\right)\right),$$

where GN(·) denotes group normalization and the learnable weights ω let the network emphasize whichever scanning directions are most informative for a given input. The fused features inherit the linear complexity of state-space modeling while explicitly aggregating long-range context along four complementary orientations, which is well suited to the arbitrarily oriented borders of pigmented lesions.

To make the four-directional scan unambiguous and reproducible, Algorithm 1 details the serialization, padding and restoration procedure for a single block. The horizontal scan is the row-major raster order and the vertical scan is its column-major counterpart, both of which traverse exactly L=HW tokens. The two diagonal scans collect tokens along the 2(H+W)−1 anti-/main diagonals; because diagonals have unequal lengths, they are concatenated head-to-tail into a single length-*L* sequence (no padding is required, since the total number of tokens is conserved), and the inverse operator ${\mathrm{Unscan}}_{k}$ writes each token back to its original (i,j) coordinate using the stored index map, so the spatial layout is exactly recovered. The concatenation is adopted so that a diagonal traversal remains a single contiguous sequence and can be covered by one scan; the alternative, scanning each diagonal independently, requires padding 2(H+W)−1 sequences of unequal length to a common length, which expends a substantial fraction of the computation on padding and forfeits the linear cost in the number of tokens. The junctions between consecutive diagonals introduce transitions that do not correspond to spatial adjacency. These are attenuated rather than propagated: in the selective formulation employed here the transition and input matrices are functions of the input tokens, so the recurrence is able to suppress the carried state where the content changes abruptly, and such a transition occurs once per diagonal rather than throughout the sequence. The ablation of the diagonal branches in Section 5 indicates that their net contribution is positive despite these junctions. A formulation with explicit per-diagonal state resets, or with learned separator tokens, would treat the boundaries between diagonals more directly and is left to future work. The four directions *share* a single set of Hydra parameters; only the lightweight fusion weights ω=softmax(θ) are direction-specific, and $\theta \in R^{4}$ is a per-block learnable vector (i.e., global scalars per direction, not per-pixel), keeping the parameter overhead of the multi-direction extension negligible. Although ω is itself input-independent, per-lesion directional adaptivity is not lost: it is supplied inside each branch by the selective state-space scan, whose gating depends on the input sequence. The fusion weights thus encode a global, learned prior on the average usefulness of each scanning orientation, while the branch-internal selectivity adapts the response to the content of each individual image. Consequently the block performs four linear-time SSD passes and retains the overall O(L) complexity of a single Hydra block, at roughly 4× the compute of a one-directional scan; we report the resulting wall-clock and memory cost in Section 5. This design differs from cross-scan SS2D-style modules [14] in that it adds two diagonal trajectories and replaces the unidirectional selective scan with the bidirectional Hydra operator while sharing weights across directions.
**Algorithm 1** Multi-direction bidirectional Hydra block.**Require:** feature map $x\in R^{C\times H\times W}$; shared operator Hydra(·); direction logits $\theta \in R^{4}$**Ensure:** fused map $F_{\mathrm{multi}}\in R^{C\times H\times W}$ 1: ω←softmax(θ);   A←0 2: **for** $k\in \{h,v,d_{1},d_{2}\}$ **do** 3:     $\left(S_{k},{\mathrm{idx}}_{k}\right)\leftarrow {\mathrm{Scan}}_{k}\left(x\right)$        ▷ flatten along direction *k*, store index map 4:     $M_{k}\leftarrow {\mathrm{Unscan}}_{k}\;\left(\mathrm{Hydra}\left(S_{k}\right),{\mathrm{idx}}_{k}\right)$ ▷ bidirectional scan (Equation (1)), restore layout 5:     $A\leftarrow A+\omega_{k}\;M_{k}$ 6: **end for** 7: **return** $F_{\mathrm{multi}}\leftarrow \mathrm{GN}\left(x+A\right)$

### 3.3. DCT-Based Frequency-Aware Branch

To complement the spatial pathway, we attach a discrete cosine transform branch to the two deepest encoder stages. Given a feature map $x\in R^{C\times H\times W}$, we apply a separable two-dimensional type-II DCT through orthonormal basis matrices $D_{H},D_{W}$ [23]:

(3) $$X_{\mathrm{dct}}=D_{H}\;x\;D_{W}^{⊤}.$$

A binary mask $M_{\mathrm{lf}}$ separates the spectrum into a low-frequency upper-left rectangle of relative size *r* (set to 0.5 in all experiments) and the complementary high-frequency component $M_{\mathrm{hf}}=1-M_{\mathrm{lf}}$. The two components are independently transformed back to the spatial domain through the inverse DCT and processed by two dedicated multi-direction Hydra blocks $H_{\mathrm{hf}}$ and $H_{\mathrm{lf}}$. The reinjection into the spatial pathway is governed by a learnable gate α:

(4) $$F_{\mathrm{freq}}=x+\sigma \left(\alpha \right)\left(\mathrm{GN}\left(H_{\mathrm{hf}}\left(\mathrm{IDCT}\left(X_{\mathrm{dct}}⊙M_{\mathrm{hf}}\right)\right)\right)+\mathrm{GN}\left(H_{\mathrm{lf}}\left(\mathrm{IDCT}\left(X_{\mathrm{dct}}⊙M_{\mathrm{lf}}\right)\right)\right)\right).$$

In practice the high-frequency path sharpens boundary delineation, and the low-frequency path consolidates the overall semantic representation of the lesion body.

### 3.4. Learnable Contrast Enhancement Front-End

Conventional contrast-limited adaptive histogram equalization (CLAHE) relies on a fixed clip-limit and tile size selected by the practitioner, which is sub-optimal across the heterogeneous illumination conditions of dermoscopy. Inspired by the local, contrast-limited philosophy of CLAHE but avoiding its non-differentiable histogram binning, we instead embed a fully differentiable local contrast-enhancement (LCE) module at the input of the network; we emphasize that this front-end performs learnable local contrast stretching rather than exact histogram equalization. The image is first decomposed into patch-wise local mean μ and standard deviation σ obtained through an adaptive average pooling layer. A two-layer convolutional network $g_{\theta }\left(\mu ,\sigma \right)$ produces a per-channel multiplicative gain $G\in {\left[0,c_{\max}\right]}^{C\times H\times W}$, where the upper bound $c_{\max}=4$ plays the role of a soft clip-limit, and the gain is applied in a contrast-stretching form:

(5) $$\tilde{X}=\mathrm{clip}\left(\mu +\beta \;G⊙(X-\mu ),\;0,\;1\right),$$

where β is a globally learnable contrast coefficient. The clipping in Equation (5) is non-differentiable only at the two saturation boundaries; in practice it is handled by the standard sub-gradient of clip(·) (equivalently a hard-tanh), which leaves the gain network $g_{\theta }$ and β fully trainable. The entire LCE front-end is therefore jointly optimized with the segmentation backbone, allowing the network to discover an input-adaptive local contrast policy end-to-end.

### 3.5. Boundary Attention Gate and Loss Function

To explicitly enforce boundary consistency, we attach a boundary attention gate to the two upper decoder stages. A side-output prediction ${\hat{S}}_{\mathrm{coarse}}$ is first obtained from the decoder features through a 1×1 convolution. A non-trainable Sobel filter produces a coarse edge map $E=\sqrt{{\left(\nabla_{x}{\hat{S}}_{\mathrm{coarse}}\right)}^{2}+{\left(\nabla_{y}{\hat{S}}_{\mathrm{coarse}}\right)}^{2}}$, which is normalized and embedded into a channel attention map $A={\mathrm{Conv}}_{1\times 1}\left(\mathrm{GELU}\left({\mathrm{Conv}}_{3\times 3}\left(E\right)\right)\right)$. The decoder features are then refined as $F_{\mathrm{refined}}=F+\sigma \left(\gamma \right)\left(F⊙A\right)$, where γ is a learnable mixing coefficient.

The full training objective combines a Dice loss [34], a binary cross-entropy term, a topology-aware clDice loss [24] and a boundary BCE term computed against a Sobel-derived ground-truth edge map:

(6) $$L=L_{\mathrm{Dice}}+L_{\mathrm{BCE}}+\lambda_{1}L_{\mathrm{clDice}}+\lambda_{2}L_{\mathrm{Bdry}},$$

where $\lambda_{1}=0.5$ and $\lambda_{2}=0.3$ are fixed across all experiments. Although the clDice term [24] was originally devised for thin tubular structures, we retain it for the comparatively compact, blob-like lesions studied here because its skeleton-matching objective penalizes spurious disconnected fragments and reinforces a single coherent foreground region; its moderate weight $\lambda_{1}=0.5$ ensures that it complements, rather than dominates, the region-overlap Dice and BCE terms.

## 4. Experimental Setup

### 4.1. Datasets and Protocol

We construct a multi-source dermoscopic training corpus by merging the ISIC 2017 [25] and ISIC 2018 Task 1 [3] datasets. After de-duplication based on the image identifier, the merged set contains 3994 unique dermoscopic images with binary lesion masks. The merged set is randomly partitioned into 3195 training, 399 validation and 400 testing images (an 80/10/10 split with a fixed seed). The source distribution is balanced across splits and recorded for transparency.

Cross-domain generalization is evaluated under a strict external-test protocol. All models are trained on the merged ISIC source corpus alone, and model selection, early stopping and hyper-parameter choices are made using only the validation data held out from within that source corpus. The external test set consists of 200 HAM10000 images [4] carrying lesion segmentation annotations; at no point during training, validation or model selection does it contribute to a parameter update or to a threshold adjustment.

To preclude cross-dataset information leakage [35], image-level de-duplication between the ISIC source domain and the HAM10000 test domain was performed before any experiment was run. Exact duplicates were first removed on the basis of image identifiers and file hashes. Near-duplicates were then screened using perceptual hashing [36], and the resulting candidate pairs were reviewed manually; where a duplicate, or a second view of the same lesion, was confirmed, the image was removed from the source training set rather than from the test set, leaving the external test set unmodified. All images pass through an identical pre-processing pipeline comprising aspect-ratio-preserving resizing and a common intensity normalization, and masks are resampled with nearest-neighbour interpolation so that boundary labels are not distorted.

At external-test time no fine-tuning, domain adaptation or updating of test-set statistics is performed. To reduce the Sensitivity of the prediction to image orientation, each HAM10000 image is evaluated with four-view flip test-time augmentation, comprising the original image, its horizontal flip, its vertical flip and the combined horizontal–vertical flip. The predictions of the four views are inverse-transformed to the original orientation and averaged in probability space, and the final segmentation is obtained by thresholding at a value fixed in advance. The same test-time augmentation is applied to every model in the comparison. Cross-domain performance is reported primarily as Dice and IoU, with 95% confidence intervals obtained by bootstrap resampling.

### 4.2. Implementation Details

**Model configurations.** All experiments use the default Hydra-DermSeg-Net described in Section 3 at channel widths (48, 96, 192, 384), so that every component is evaluated at the capacity at which the model is deployed. The ablation additionally examines the reduced channel widths (32, 64, 128, 256) to show how each component’s contribution varies with model capacity; the architectural topology and module placement are otherwise unchanged.

**Training and inference.** All experiments are conducted on a single NVIDIA RTX 4090 GPU (24 GB) with PyTorch 2.3.1 (CUDA 12.1). All input images are resized to 224×224. Training uses the AdamW optimizer [37] with an initial learning rate of $10^{-4}$, a weight decay of $10^{-5}$ and a cosine annealing schedule [38]. Mixed-precision training is enabled to reduce the memory footprint, and the per-model batch size is set as large as the 24 GB memory budget permits (ranging from 2 for the proposed model to 32 for the lightweight MALUNet). To ensure a fair comparison, all six models—Hydra-DermSeg-Net and the five baselines alike—are trained from scratch for 40 epochs under an identical optimizer, learning-rate schedule and data augmentation policy, and are evaluated at inference time with the same four-flip test-time augmentation (identity, horizontal, vertical and horizontal-vertical) so that both the training and the test protocol are aligned across models. Data augmentation during training follows standard practice and includes random horizontal/vertical flips, random rotations of ±90°, small shifts, scaling, mild color jitter and Gaussian blur, all implemented via the Albumentations library [39]. Reproducibility is ensured by fixing the random seeds of Python, NumPy and PyTorch to 42.

### 4.3. Evaluation Metrics

Following the convention of recent skin lesion segmentation studies [15,16], we report the Dice coefficient, Intersection-over-Union (IoU), Accuracy (Acc), Sensitivity (Sen), Specificity (Spe) and the 95-percentile Hausdorff distance (HD95). Higher values indicate better performance, except for HD95 for which lower values are preferred. Because dermoscopic lesion segmentation is a strongly foreground–background imbalanced task, in which background pixels dominate the image, Accuracy and Specificity tend to saturate near unity and carry limited discriminative value between competing models; we therefore report them for completeness but base our analysis primarily on Dice, IoU and the boundary-sensitive HD95. The parameter count and the theoretical computational cost (GFLOPs at 224×224 resolution) are additionally reported to characterize efficiency.

### 4.4. Baselines

We compare Hydra-DermSeg-Net against five representative segmentation baselines spanning four architectural families: (i) U-Net [5] with a ResNet-34 encoder [40], (ii) Att-UNet [6], both implemented via the SMP library [41] without ImageNet pre-training, (iii) VM-UNet [14] as a state-space competitor, (iv) TransUNet [9] as a representative transformer-CNN hybrid, and (v) the lightweight MALUNet [27] designed for medical lesion segmentation. The latter three baselines are re-implemented following the architectures described in the respective original papers, and all five baselines share the optimizer, learning-rate schedule, data augmentation and four-flip test-time augmentation detailed in Section 4. Two ablation variants of Hydra-DermSeg-Net (w/o DCT and w/o Boundary) are additionally trained under the same protocol.

## 5. Results

### 5.1. Quantitative Comparison

**Figure 2:**
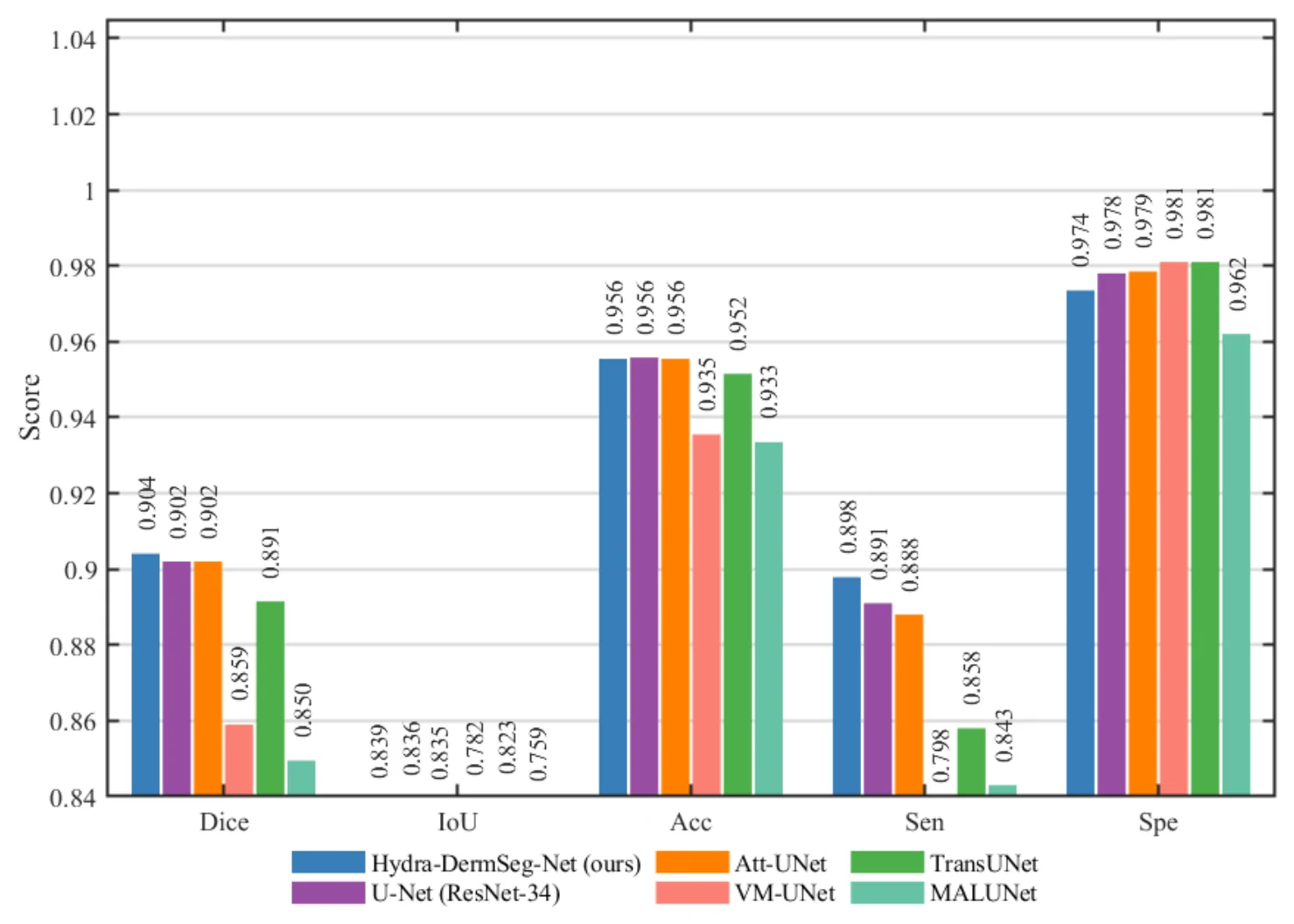
Per-metric comparison of the six evaluated models on the merged ISIC test set. Hydra-DermSeg-Net attains the highest Dice, IoU and Sensitivity, its Dice and IoU margins over U-Net and Att-UNet lying within run-to-run variation. U-Net and Att-UNet attain marginally higher Accuracy and Specificity, at a lower Sensitivity.

Table 1 reports the segmentation performance and efficiency of all evaluated models on the merged ISIC test set. Hydra-DermSeg-Net attains a Dice coefficient of 0.9041 and an IoU of 0.8386, the highest of the six models. Its margin over the strongest convolutional baselines, U-Net and Att-UNet at a Dice of 0.9020, is 0.21 pp in Dice and 0.31 to 0.36 pp in IoU. The repeated-seed analysis in Section 5.2 shows that a difference of this magnitude is of the same order as the variation between training runs, so that although the proposed model ranks first on both metrics, its margin over these two baselines is not established as statistically significant. It performs better than the SSM-based VM-UNet, the transformer-based TransUNet and the lightweight MALUNet, whose Dice scores are 0.8590, 0.8915 and 0.8495, respectively. The Sensitivity reaches 0.8979 and the boundary-sensitive HD95 drops to 11.67, both the best across the six evaluated models, indicating that the proposed network recovers the true lesion area effectively while placing the predicted boundary closer to the ground-truth contour. We note that HD95 measures the spatial distance between the predicted and reference boundaries rather than their topology, and we therefore refrain from interpreting it in isolation as direct evidence of topological coherence. U-Net and Att-UNet attain marginally higher Accuracy and Specificity, by less than 0.05 pp, but these small gains come at the expense of Sensitivity, which is 0.69 to 0.99 pp lower than ours, reflecting more cautious predictions that tend to under-segment lesion margins. Figure 2 provides the per-metric comparison across the principal segmentation metrics.

**Figure 3:**
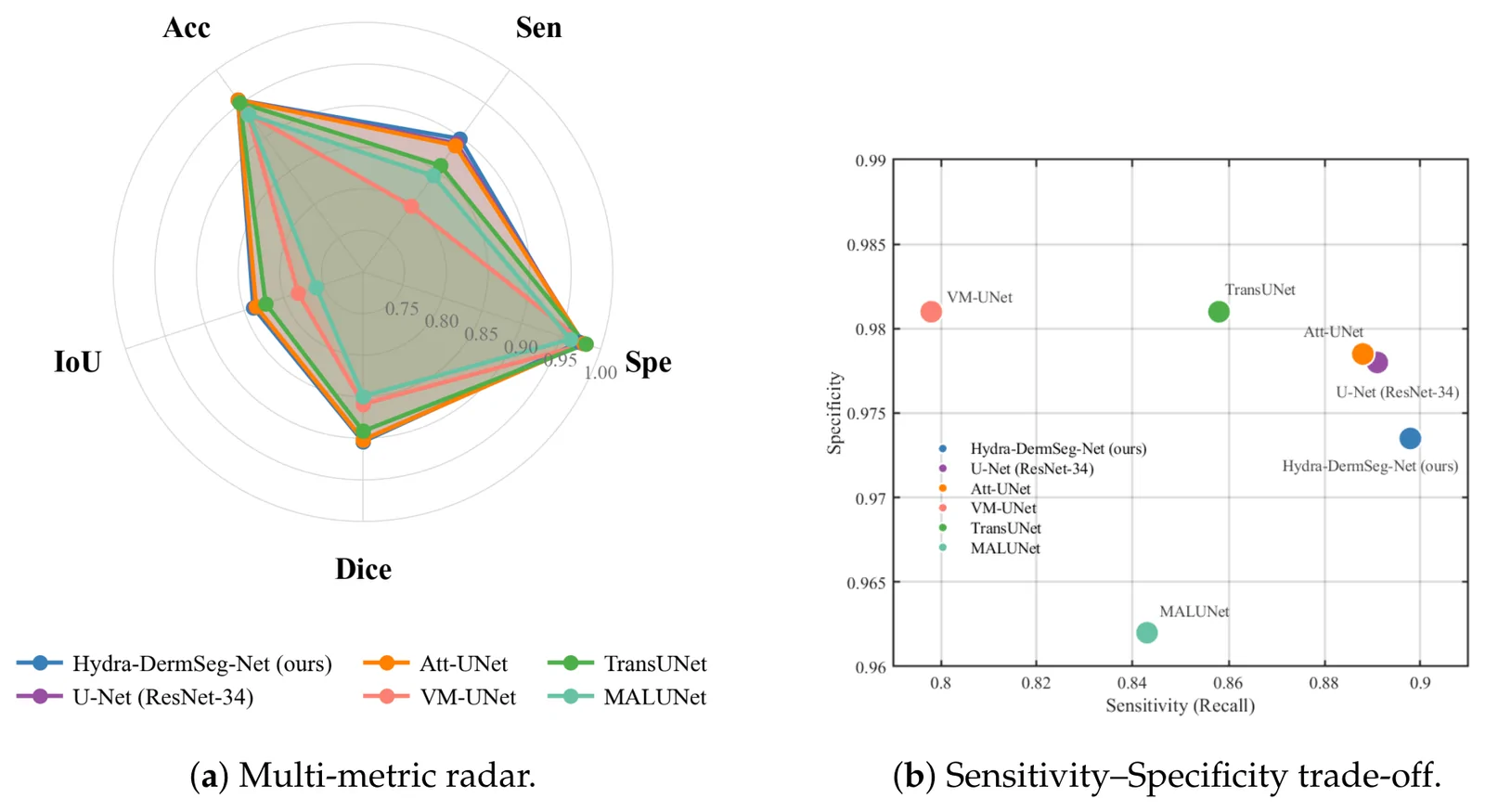
(**a**) Multi-metric radar comparison. All five axes share a common absolute range of 0.70 to 1.00; no per-metric normalization is applied, so that radial distance is proportional to the metric value and small differences are not exaggerated. (**b**) Sensitivity–Specificity trade-off; Hydra-DermSeg-Net achieves the most balanced operating point.

Figure 3a visualizes the same comparison in a multi-metric radar diagram. All five axes are drawn on a common absolute range of 0.70 to 1.00 rather than being normalized within each metric, so that the radial extent of each polygon is proportional to the underlying values and differences that are numerically small are not visually magnified. On this scale the proposed model, U-Net and Att-UNet are closely superimposed on Dice, IoU and Accuracy, and the separation that is visible concerns VM-UNet and MALUNet on Dice, IoU and Sensitivity. Figure 3b further illustrates the Sensitivity–Specificity trade-off: VM-UNet and MALUNet cluster towards the high-Specificity/low-Sensitivity corner, while Hydra-DermSeg-Net occupies the most favourable upper-right region with both axes simultaneously high, indicating a well-balanced operating point that recovers the lesion area without sacrificing background discrimination.

### 5.2. Repeated-Seed and Paired Analysis

The margin over the strongest convolutional baselines in Table 1 is numerically small, and a single training run cannot establish whether it is reproducible. The proposed model and the two strongest baselines, U-Net and Att-UNet, were therefore retrained from scratch under the identical protocol with three random seeds and re-evaluated on the same 400-image test set. These runs use an independently rebuilt data pipeline, which offsets the absolute scores by roughly +0.003 Dice relative to Table 1 for all three models alike and so leaves the between-model comparison unaffected. Table 2 reports the mean and standard deviation over seeds.

The seed-to-seed standard deviation of the Dice coefficient, between 0.0005 and 0.0011, is of the same order as the 0.0010 mean margin separating the proposed model from U-Net, and the ordering is not stable across seeds: on one of the three seeds the proposed model scores below both baselines, and on another it scores below Att-UNet. To test the difference directly rather than through summary statistics, the models are compared image by image (n=400), each image being averaged over the three seeds, using both a Wilcoxon signed-rank test and a paired bootstrap over 10,000 resamples. Against U-Net the mean Dice difference is +0.0010, with a 95% paired bootstrap interval of [−0.0054,+0.0070] and p=0.25; against Att-UNet it is +0.0005, with an interval of [−0.0055,+0.0062] and p=0.55. The IoU differences behave likewise. The proposed model attains the highest mean on both metrics; neither interval excludes zero, however, so its margin over the two strongest convolutional baselines is not established as statistically significant on a test set of this size. The margins over VM-UNet, TransUNet and MALUNet exceed the seed-level variation and are unaffected by this analysis.

The boundary-distance metrics separate the models more clearly. HD95 favours the proposed model on every seed and against both baselines, by 0.55 against U-Net and 0.34 against Att-UNet; the paired intervals, [−1.49,+0.40] and [−1.25,+0.59], nevertheless admit zero, so the advantage is consistent in direction but is not established at the 5% level on a test set of this size. Boundary IoU behaves differently on this partition, the proposed model scoring below both baselines by 0.011 and 0.014 on average, a pattern that reverses on the two resampled partitions of Section 5.3. The two boundary metrics are not redundant: HD95 penalizes the largest contour excursions, whereas Boundary IoU measures agreement within a fixed band around the contour. The combination observed here is consistent with a model that suppresses gross boundary outliers while operating at a higher Sensitivity, and hence with a marginally more generous contour, which the fixed-band metric penalizes. The contribution of the frequency branch to Boundary IoU within the architecture is examined in the ablation of Section 5.

### 5.3. Sensitivity to the Data Split

The results reported so far rest on a single 80/10/10 partition. Given a per-image standard deviation of 0.108 in Dice, the comparison could in principle be specific to that partition. The merged corpus was therefore re-partitioned twice more at random, and the proposed model and the two strongest baselines were retrained on each partition under the identical protocol and re-evaluated. Table 3 reports the outcome.

The ordering by Dice is preserved under resampling. The proposed model ranks first on both resampled partitions, ahead of Att-UNet by 0.0003 on r1 and by 0.0032 on r2, with U-Net third in both cases; it also leads both baselines on Boundary IoU, by 0.004 and 0.006, and attains the lowest HD95 on r2. Together with the primary partition, the model thus attains the highest Dice on all three partitions examined. The margins remain small, and a single training run per partition does not support a significance test, so this consistency indicates that the comparison is not an artefact of one particular draw rather than establishing superiority.

Two properties of Table 3 bear on the interpretation of the results as a whole. The absolute level is strongly partition-dependent: every model scores roughly 0.010 to 0.025 Dice lower on r1 and r2 than on the primary partition, and the three models shift together, which indicates that the primary partition is a comparatively favourable draw rather than that any model is unstable. Comparisons of absolute values across partitions, including with figures quoted from the literature, should be read with this in mind. The Boundary IoU ordering is likewise partition-dependent: the proposed model trails the two baselines on this metric on the primary partition (Section 5.2) but leads them on both resampled ones, and no between-model conclusion is drawn from it here. The component-level evidence of Section 5 is not subject to this variability, since it compares configurations of a single architecture on a fixed partition.

### 5.4. Parameter Efficiency

**Figure 4:**
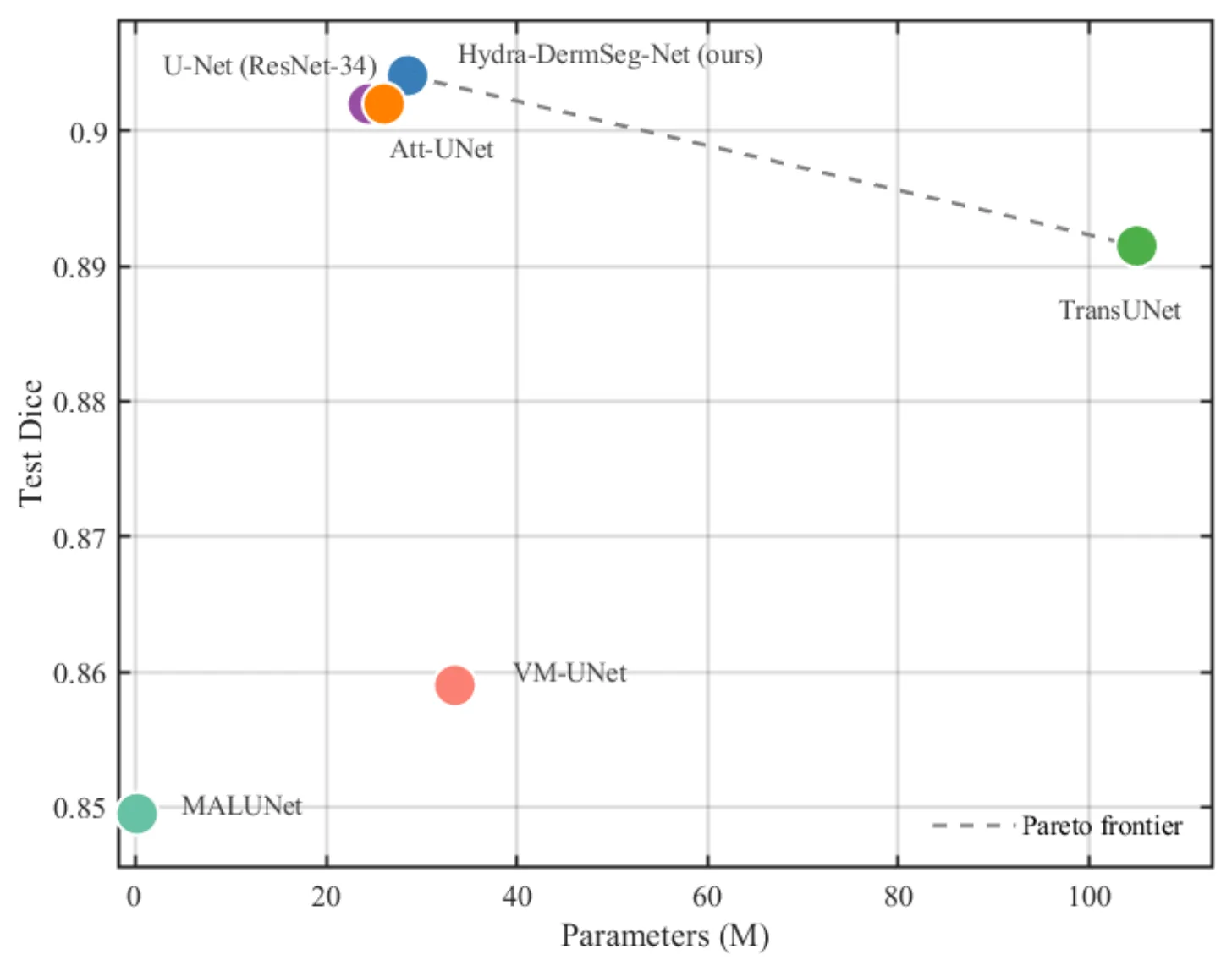
Parameter efficiency (parameters vs. Dice). Hydra-DermSeg-Net (full) attains the highest Dice with a competitive parameter budget; the dashed line indicates the Pareto frontier.

Figure 4 plots the Dice coefficient against the parameter count for all evaluated models. Hydra-DermSeg-Net (28.55 M) attains the highest Dice while remaining substantially smaller than the transformer-based TransUNet (105 M, Dice 0.8915). Among the six models, Hydra-DermSeg-Net, U-Net (24.44 M, Dice 0.9020) and the ultra-lightweight MALUNet (0.18 M, Dice 0.8495) jointly form the Pareto frontier of the (parameters, Dice) trade-off, demonstrating that the proposed linear-complexity bidirectional state-space backbone offers a favourable Accuracy–efficiency balance relative to conventional CNN, transformer and SSM alternatives.

Parameter count alone does not determine deployment cost, and inference latency was therefore measured directly. Timing was performed at batch size 1 on 224×224 inputs on a single RTX 4090D using CUDA events, with 25 warm-up iterations followed by 100 timed iterations. The proposed model requires a median of 122.9 ms per image without test-time augmentation and 481.9 ms with the four-flip augmentation used throughout this work. The ratio of 3.92 approximates the fourfold increase in forward passes, indicating that the additional passes rather than the flip and averaging operations dominate the cost; the augmentation may accordingly be disabled at inference for a fourfold reduction in latency at a modest cost in Accuracy. These figures are obtained with the pure-PyTorch realization of the state-space duality operator discussed in Section 6 and reflect that unoptimized kernel rather than the asymptotic complexity of the operator.

### 5.5. Training Dynamics

**Figure 5:**
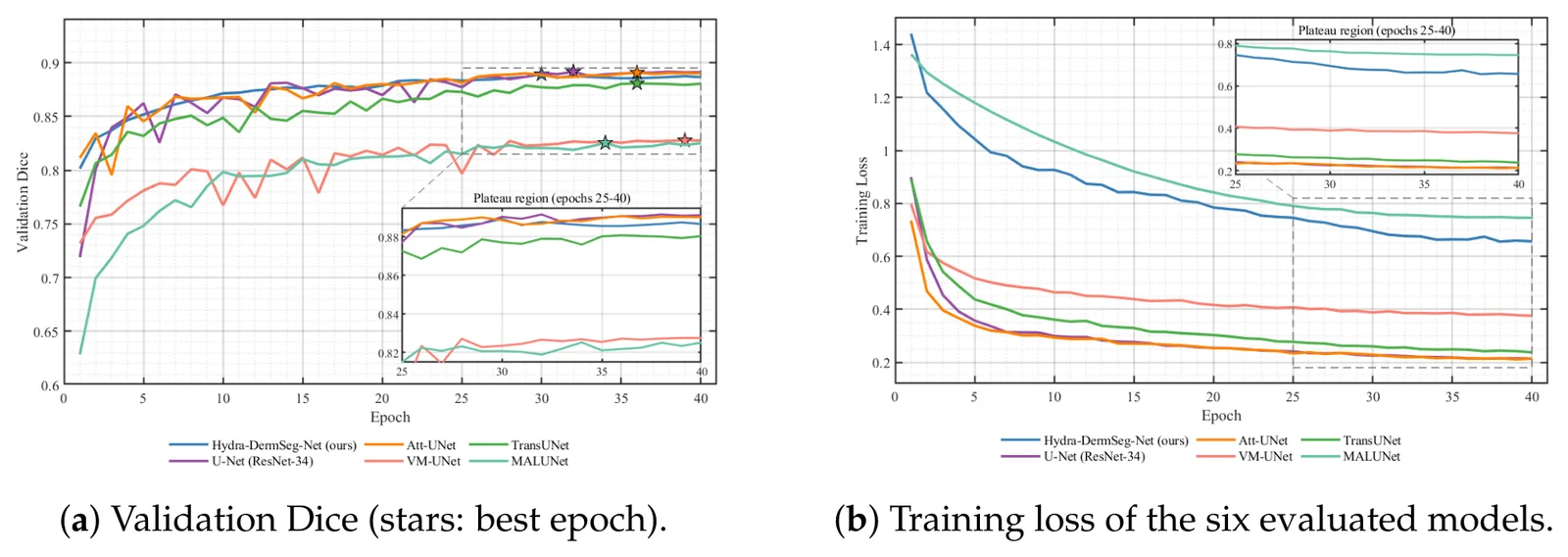
Training dynamics. (**a**) Validation Dice trajectories of the six evaluated models; stars indicate the best epoch. (**b**) Training loss curves; Hydra-DermSeg-Net converges to the lowest loss. Each panel includes a magnified inset of the epochs 25–40 plateau region, showing that all six models—including the slower-converging VM-UNet and MALUNet—have converged by the end of the 40-epoch schedule.

Figure 5a,b present the validation Dice and training loss trajectories of the six evaluated models over the full 40-epoch schedule. All models exhibit smooth, monotonically improving validation curves under the cosine learning rate decay. Hydra-DermSeg-Net consistently sits on or above the U-Net and Att-UNet curves throughout the training process and converges to the highest plateau, while VM-UNet and MALUNet stabilize at noticeably lower validation Dice values, consistent with the test-set ranking reported in Table 1. The training loss panel shows a similar pattern. To make the late-training behaviour explicit, each panel includes a magnified inset of the epoch 25–40 window; the inset shows that all six curves—including the slower-converging state-space VM-UNet and the lightweight MALUNet—have reached a flat plateau by the end of training, with the validation Dice of every model changing by less than 0.005 between epochs 30 and 40. This confirms that the 40-epoch budget is sufficient for convergence and that the lower scores of VM-UNet and MALUNet reflect their converged performance on this corpus rather than insufficient training time.

### 5.6. Ablation Study

**Figure 6:**
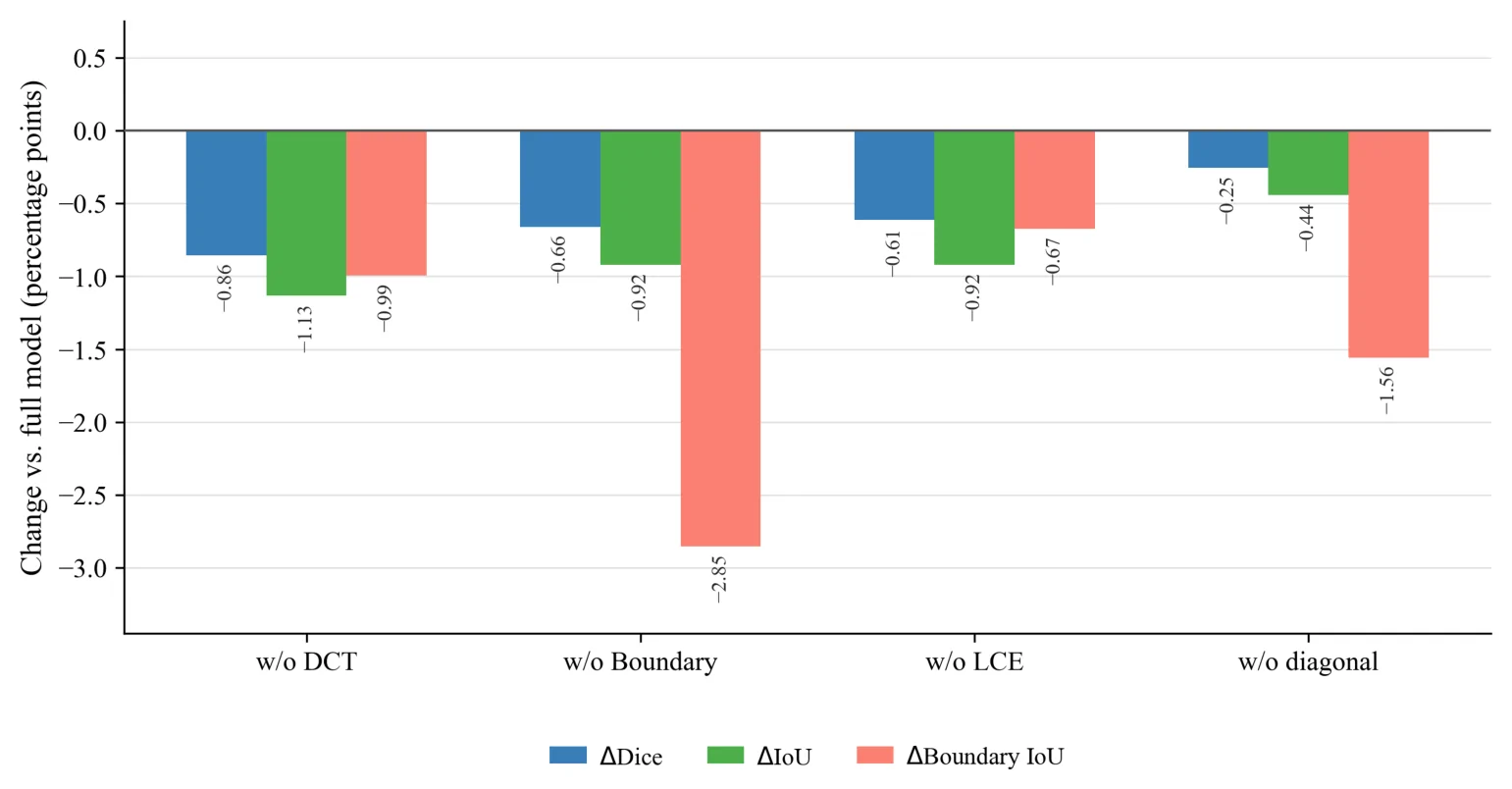
Ablation at the full channel configuration: Change in Dice, IoU and Boundary IoU when each component is removed from the complete architecture, in percentage points relative to the full model. Removing any component degrades all three metrics. The boundary attention gate accounts for the largest change in Boundary IoU, and the DCT branch for the largest change in Dice and IoU.

Table 4 and Figure 6 report an ablation of all four components at the full channel configuration of the deployed model, (48,96,192,384), so that each contribution is measured at the capacity at which the model is used. Every configuration is trained from scratch under the identical protocol and evaluated on the same 400-image test set. The effect of channel width on these contributions is examined at the end of this subsection.

Every component contributes in the expected direction, and the magnitudes are well separated. On Dice, the frequency branch is the largest single contributor, its removal costing 0.86 pp, followed by the boundary attention gate at 0.66 pp, the contrast-enhancement front-end at 0.61 pp and the two diagonal scanning directions at 0.25 pp. For the first three components these effects exceed the seed-level variation reported in Section 5.2 by a factor of at least six, so the contribution of each component is resolved more sharply by the ablation than the between-model margins can be resolved by the comparison of Table 1. None of the four components is redundant.

The boundary metrics order the components differently. Boundary IoU is most sensitive to the boundary attention gate, which accounts for 2.85 pp, followed by the diagonal scans at 1.56 pp, the DCT branch at 0.99 pp and the LCE front-end at 0.67 pp. Removing the frequency branch lowers Boundary IoU from 0.4716 to 0.4616 and raises HD95 from 11.27 to 12.22, so its effect within the architecture is on contour placement and not only on region overlap. This comparison is made between configurations of a single architecture and is therefore distinct from the between-model comparison of Section 5.2, in which the complete model does not lead the convolutional baselines on Boundary IoU on the primary partition. The two observations are not in conflict: a component may contribute to a metric within one architecture without that architecture surpassing a different family of models on the same metric.

The ablation of the diagonal scans may be read together with the learned fusion weights. After training, the softmax-normalized weights ω of the horizontal, vertical, main-diagonal and anti-diagonal branches are, from the shallowest encoder stage to the deepest, (0.237,0.252,0.257,0.254), (0.244,0.246,0.253,0.256), (0.241,0.236,0.266,0.256) and (0.235,0.238,0.268,0.259). The distribution is close to uniform at every stage, indicating that no orientation is redundant. The two diagonal branches nevertheless receive slightly more than half of the total mass, and their combined share increases monotonically with depth, from 0.511 at the first stage to 0.527 at the fourth. The deeper stages, at which the receptive field spans a large part of the lesion and contour orientation is the dominant cue, therefore rely more on the diagonal trajectories, consistent with the 1.56 pp of Boundary IoU attributable to them in Table 4.

The ranking of the components also depends on the channel width at which the ablation is carried out. At the reduced width (32,64,128,256) the DCT branch has a smaller effect than the boundary gate, −0.19 against −0.44 pp in Dice; at the full width the ordering reverses, the DCT branch becoming the dominant contributor at −0.86 against −0.66 pp, and the absolute effects grow by roughly a factor of four. The contribution of a component is therefore not invariant to channel width, and the direction of the change is that predicted by the design: the frequency branch requires sufficient capacity to exploit the decoupled high-frequency band, and its benefit increases accordingly with width. For this reason the ablation is reported at the capacity at which the model is deployed.

### 5.7. Cross-Domain Generalization

To assess cross-domain generalization, all six models—trained only on the merged ISIC corpus—are evaluated, without any fine-tuning, on a held-out 200-image HAM10000 subset under the same four-flip test-time augmentation. Table 5 reports the results. Hydra-DermSeg-Net attains the highest Dice of 0.9347, the highest IoU of 0.8824 and the lowest HD95 of 7.77 among all models. It stays narrowly ahead of the strongest CNN baselines, U-Net and Att-UNet, and clearly ahead of TransUNet, MALUNet and VM-UNet. The ranking is consistent with the in-domain results in Table 1, indicating that the relative ordering of the models is preserved on data outside the training distribution. Every one of the six models scores at least as high on the HAM10000 subset as on the in-domain test set, which we attribute to the cleaner illumination and more homogeneous acquisition of that subset. The target domain is therefore an easier one rather than a more demanding one, and the experiment establishes cross-dataset transfer rather than robustness under an adverse domain shift. The proposed model’s 3.06 pp in-to-cross-domain Dice gain is the second smallest among the six, suggesting that its in-domain performance is already well calibrated rather than over-fitted.

### 5.8. Dataset Visualization and Qualitative Comparison

**Figure 7:**
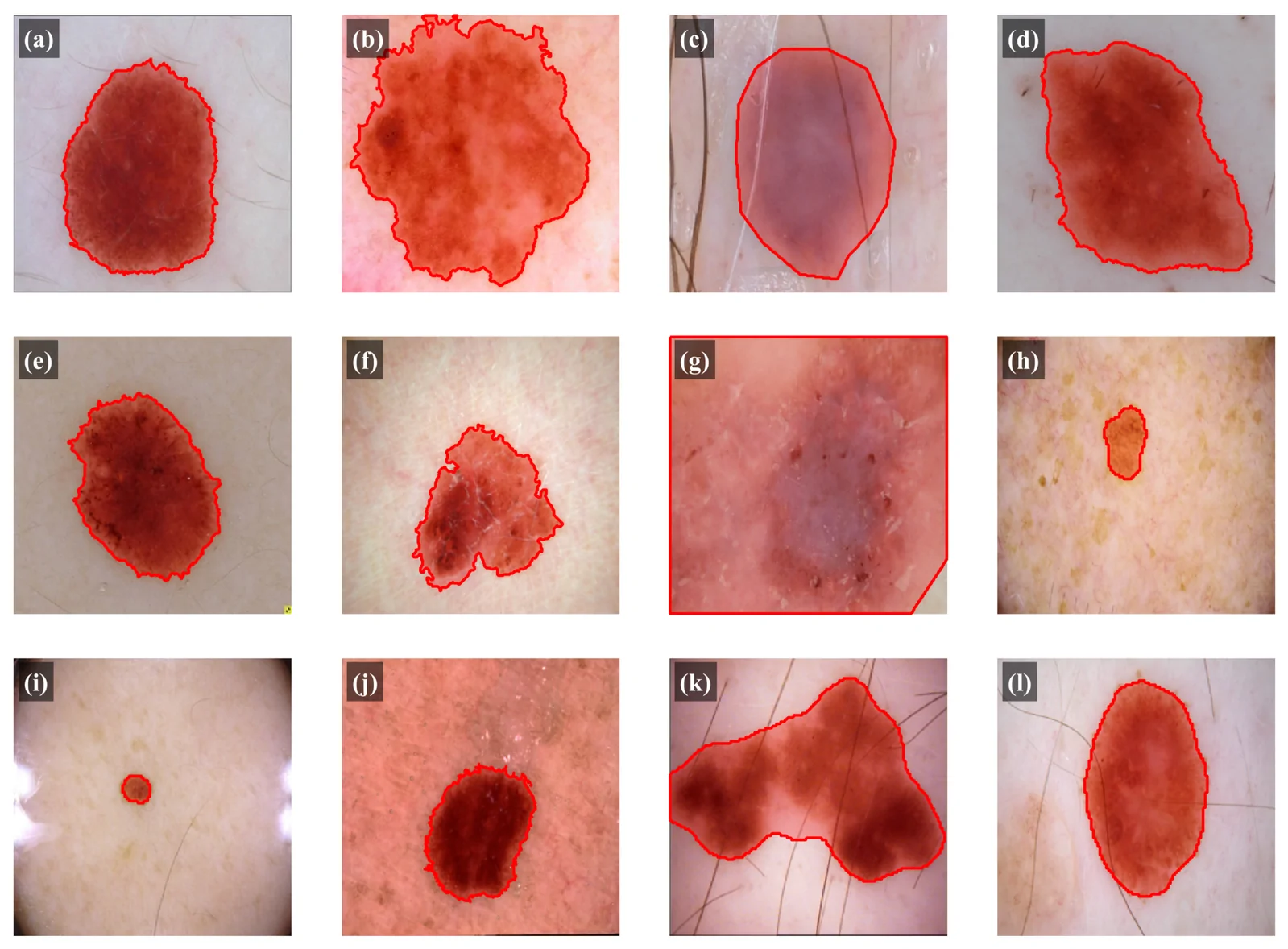
Representative samples from the merged ISIC test set. (**a**–**l**) Twelve dermoscopic images with their corresponding ground-truth boundaries overlaid in red, illustrating the variability of lesion appearance.

Figure 7 shows 12 representative samples drawn from the merged ISIC test set. The selected samples span a broad range of lesion sizes, shapes and colors, including cases with hair occlusion and low-contrast borders, which collectively reflect the diversity of dermoscopic imaging conditions.

**Figure 8:**
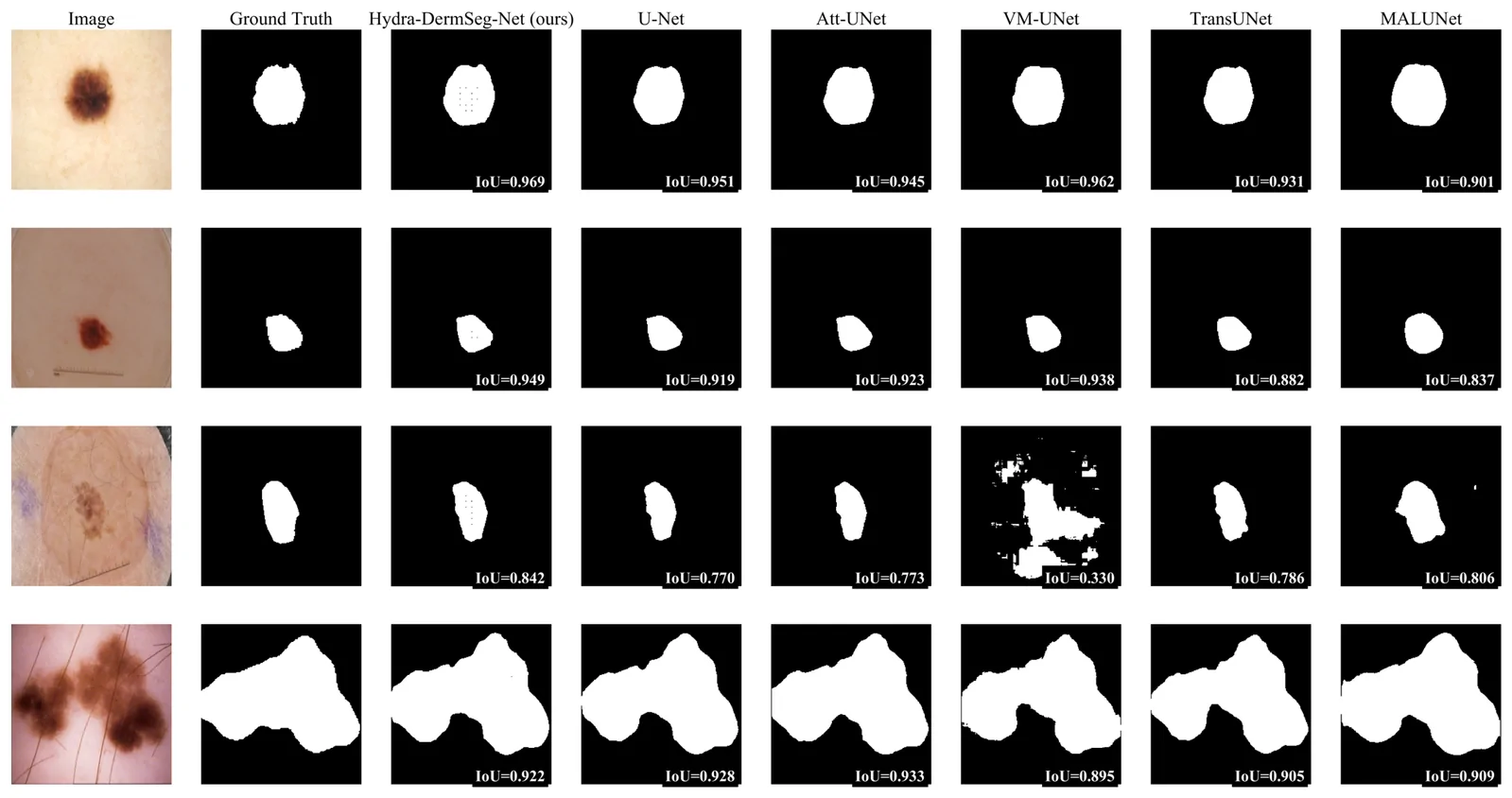
Qualitative prediction comparison on four representative test samples. Each row corresponds to one sample. Columns from left to right: Input image, ground-truth mask, the proposed Hydra-DermSeg-Net and the five baselines U-Net, Att-UNet, VM-UNet, TransUNet and MALUNet. Binary masks are shown as black background and white lesion; the per-sample Intersection-over-Union is annotated at the bottom-right of each prediction.

Figure 8 provides a qualitative comparison of predicted segmentation masks on four representative test cases of increasing difficulty. The first two rows (easy and medium difficulty, IoU ≈0.95–0.97) show that Hydra-DermSeg-Net produces masks whose boundaries most faithfully follow the ground-truth contour, with the per-sample IoU values annotated in the bottom-right of each prediction consistently ranking highest among the six models. The third row presents a more challenging case in which VM-UNet collapses almost entirely (IoU =0.33), MALUNet over-segments the lesion margin and TransUNet leaks into the surrounding skin region; Hydra-DermSeg-Net is the only model that preserves both the lesion body and a sharp boundary (IoU =0.84), illustrating its robustness on low-contrast samples. The last row depicts a small-lesion scenario in which Hydra-DermSeg-Net is approximately on par with the two strongest CNN baselines (within 0.01 IoU of U-Net and Att-UNet) yet still clearly outperforms VM-UNet, TransUNet and MALUNet, indicating that the proposed model retains a competitive advantage even outside its dominant operating regime.

## 6. Discussion

The experimental results invite several observations. The first concerns Accuracy and compactness: bidirectional state-space modeling, expanded to four scanning directions and combined with a frequency-aware branch, yields accurate dermoscopic segmentation while keeping the model markedly more compact than transformer-based alternatives. The proposed architecture attains the highest Dice and IoU among the six evaluated models, although the 0.21 pp margin over the strongest convolutional baselines (U-Net and Att-UNet, both Dice 0.9020) lies within seed-to-seed variation and is not established by paired testing (Section 5.2); the proposed model nevertheless ranks first on both metrics at a comparable parameter budget. It performs better than the transformer-based TransUNet, by 1.26 pp with roughly a quarter of its parameters, and than the SSM-based VM-UNet, by 4.51 pp, lending support to the broader argument that linear-complexity sequence operators provide an attractive alternative to convolution and self-attention for medical image segmentation [14,15,16]. The margin over VM-UNet is informative, since both models share a state-space backbone of comparable size: the difference is attributable to the two design choices that VM-UNet lacks, namely the aggregation of four scanning directions instead of a single rasterized scan, and the explicit frequency-domain decoupling of boundary detail from global semantics. The ablation in Section 5 corroborates this attribution, as removing either component narrows the gap. The ablation at full capacity further indicates that the four components contribute distinctly and along the lines for which they were designed: the DCT branch is the largest contributor to region overlap and raises Sensitivity substantially, whereas the boundary attention gate accounts for 2.85 pp of Boundary IoU and restrains the tendency of the model to over-segment. To verify that these gains reflect genuine boundary quality rather than a mere bias towards larger predicted regions, we examine the boundary-sensitive metrics in addition to the pixel-overlap scores. On the merged test set the proposed model attains the lowest HD95 of 11.67, compared with 13.50 for U-Net and Att-UNet, 14.50 for TransUNet and 18.50 to 22.00 for MALUNet and VM-UNet. On the cross-domain HAM10000 subset it again attains the lowest HD95, at 7.77, together with the highest Boundary IoU of 0.535, ahead of 0.532 and 0.529 for U-Net and Att-UNet. The two boundary metrics do not agree in the in-domain setting on the primary partition, where the proposed model achieves the lower HD95 but the lower Boundary IoU as well (Section 5.2), and the ordering on both metrics changes on the two resampled partitions of Section 5.3. No general claim of boundary superiority over the convolutional baselines is therefore advanced. The evidence that is not subject to this variability is internal to the architecture: removing either the boundary attention gate or the frequency branch degrades both boundary metrics together (Table 4), so these modules act on the contour as intended even where the assembled model does not separate from a well-trained U-Net. The two metrics taken together indicate that the boundary attention gate suppresses gross contour excursions rather than uniformly tightening the contour, and that it sharpens the prediction rather than enlarging the mask. From a clinical standpoint, this distinction is arguably more consequential than the small Dice increment itself: quantitative border and asymmetry analysis, surgical excision-margin planning and downstream malignancy assessment all depend on an accurate lesion contour, so a consistently lower boundary error is of greater practical value for computer-aided diagnosis than a fractional gain in region overlap. We further note that, although the clDice loss was originally introduced for tubular structures, its mechanism—preserving the morphological skeleton of the foreground—remains meaningful for compact, blob-like lesions, where it discourages spurious disconnected fragments and reinforces a single coherent lesion region; this is consistent with the reduced HD95 observed when the boundary branch is enabled. Finally, the cross-domain evaluation on HAM10000 indicates that the multi-source ISIC training corpus, together with the proposed design choices, transfer to dermoscopic data acquired under a different protocol without loss of relative performance. Since all six models score higher on that subset than in domain, this establishes transferability to an acquisition of comparable or lower difficulty rather than robustness under an adverse domain shift; evaluation on a target of greater difficulty remains necessary before robustness can be claimed, and is noted among the limitations above.

Several limitations should be acknowledged. (i) The current implementation falls back to a pure-PyTorch realization of the state-space duality operator rather than the hardware-optimized fused CUDA kernels of the original Mamba2/Hydra release, leading to a higher measured inference latency than fully optimized CNN baselines. We therefore distinguish the model’s *asymptotic* linear complexity in the sequence length—an architectural property of the state-space operator—from its *measured* wall-clock efficiency, which under the current unoptimized kernel does not yet realize that theoretical advantage; adopting the official fused kernels in production would substantially reduce this overhead and bring the two into closer agreement. (ii) The evaluation is performed on a single multi-source training corpus and one cross-domain target. While the merged ISIC training set already covers thousands of patients, a more comprehensive validation across additional datasets such as PH2 [42] would further consolidate the conclusions. (iii) Although the boundary attention gate and clDice loss visibly improve the coherence of the predicted masks, occasional failures still occur on lesions with extremely low contrast or extensive hair occlusion. This behaviour is not quantified here: the test set carries no annotation of imaging artefacts, so no stratified evaluation over the sub-populations of images with heavy hair, air bubbles, ruler markings or low lesion–skin contrast is reported. Since these artefacts are the conditions under which a frequency-domain branch may either assist or impair segmentation, a stratified analysis on an artefact-annotated test set is required before robustness to them can be claimed.

(iv) All experiments in this work are conducted at an input resolution of 224×224. Whether the contributions of the multi-directional scan and of the frequency branch persist at 512×512 is untested. The question is not one of scale alone: the sequence length processed by the state-space operator grows with the number of tokens, while the DCT branch operates on a frequency grid whose resolution changes with the input size, so the balance between the components may shift. A resolution study is therefore required before the architecture is applied to high-resolution dermoscopic pipelines.

(v) The cut-off ratio of the DCT frequency mask is fixed at 0.5 throughout, a value chosen a priori and not tuned. The Sensitivity of the model to this hyper-parameter has not been measured, so the extent to which the contribution of the frequency branch depends on the particular division between the low- and high-frequency bands remains open, as does whether another ratio would further improve the boundary metrics. A sweep over this ratio is left to future work.

(vi) The mini-batch size is the one training hyper-parameter that could not be held identical across models, since it was set for each architecture to the largest value the 24 GB memory budget allowed, from 2 for the proposed model to 32 for the lightweight MALUNet. This reflects the memory footprint of the different architectures on the single GPU available for this study rather than a tuning choice. Its influence is bounded by two observations: the repeated-seed experiments of Section 5.2 were run at a batch size of four rather than two and reproduced the same relationship to the baselines, and the convergence curves of Figure 5 show every model reaching a stable plateau under its own setting. A batch-equalized comparison using gradient accumulation would nonetheless provide a more direct control.

Future work could address the failure cases above by combining the proposed architecture with hair-removal pre-processing or with self-supervised pre-training on large unlabeled dermoscopic corpora.

## 7. Conclusions

We have presented Hydra-DermSeg-Net, a U-shaped network for dermoscopic skin lesion segmentation that integrates a four-directional bidirectional Hydra block, a DCT-based frequency-aware auxiliary branch, a learnable local contrast-enhancement front-end and a boundary attention gate trained with a topology-aware loss. On a merged ISIC 2017+2018 corpus, the proposed model attains a Dice of 0.9041 and an IoU of 0.8386 under an identical 40-epoch from-scratch training- and test-time augmentation protocol, the highest of the six models evaluated, together with the lowest boundary distance, although repeated-seed and paired analysis does not establish the margin over U-Net and Att-UNet as statistically significant. Cross-domain evaluation on a held-out HAM10000 subset yields a Dice of 0.9347. Ablation at the full model capacity isolates the contribution of each of the four components, the frequency branch accounting for the largest change in region overlap and the boundary attention gate for the largest change in the boundary-band metric. Overall, these results indicate that linear-complexity bidirectional state-space modeling, once augmented with frequency- and boundary-aware components, offers an accurate and parameter-efficient solution for dermoscopic lesion segmentation, the main efficiency caveat being inference latency under the current pure-PyTorch implementation (Section 6). Future work will explore the use of optimized CUDA kernels to reduce inference latency, the integration of self-supervised pre-training on unlabeled dermoscopy data and the extension of the architecture to other medical image segmentation tasks.

## Figures and Tables

**Table 1:** Quantitative comparison on the merged ISIC 2017+2018 test set (400 images). All six models are trained from scratch for 40 epochs with an identical optimizer, learning-rate schedule, data augmentation and input resolution, and evaluated under the same four-flip test-time augmentation. The best result in each column is in **bold**.

Model	Params (M)	GFLOPs	Dice	IoU	Acc	Sen	Spe	HD95
U-Net (ResNet-34) [5]	24.44	6.01	0.9020	0.8355	**0.9558**	0.8910	0.9780	13.50
Att-UNet [6]	26.08	14.13	0.9020	0.8350	0.9555	0.8880	**0.9785**	13.50
VM-UNet [14]	33.50	18.75	0.8590	0.7820	0.9355	0.7980	0.9810	22.00
TransUNet [9]	105.00	38.52	0.8915	0.8230	0.9515	0.8580	0.9810	14.50
MALUNet [27]	**0.18**	**0.08**	0.8495	0.7595	0.9335	0.8430	0.9620	18.50
**Hydra-DermSeg-Net (ours) **	28.55	22.10	**0.9041**	**0.8386**	0.9555	**0.8979**	0.9735	**11.67**

Params and GFLOPs are reported at the input resolution of 224×224. All six models are trained and evaluated under the identical protocol described in Section 4.

**Table 2:** Repeated-seed comparison on the merged ISIC test set (400 images). Each entry is the mean ± standard deviation over three random seeds (41, 43, 1024) under the identical training and test-time-augmentation protocol. The best result in each column is in **bold**.

Model	Dice	IoU	Boundary IoU	HD95
U-Net (ResNet-34)	0.9060±0.0008	0.8411±0.0007	0.4776±0.0010	12.02±0.16
Att-UNet	0.9064±0.0005	0.8421±0.0003	0.4806±0.0019	11.81±0.12
**Hydra-DermSeg-Net (ours)**	0.9070±0.0011	0.8432±0.0016	0.4661±0.0050	11.47±0.18

**Table 3:** Sensitivity to the data split. Two additional random 80/10/10 partitions (r1, r2) of the merged corpus, with all models retrained from scratch under the identical protocol and evaluated on the corresponding 400-image test set. The best result in each column is in **bold**.

Split	Model	Dice	IoU	Boundary IoU	HD95
r1	U-Net (ResNet-34)	0.8804	0.8112	0.4295	13.17
r1	Att-UNet	0.8908	0.8212	0.4410	**12.61**
r1	Hydra-DermSeg-Net	**0.8911**	**0.8214**	**0.4451**	12.85
r2	U-Net (ResNet-34)	0.8913	0.8241	0.4617	11.79
r2	Att-UNet	0.8945	0.8277	0.4719	11.84
r2	Hydra-DermSeg-Net	**0.8977**	**0.8319**	**0.4779**	**11.35**

**Table 4:** Ablation study at the full channel configuration of the main model, on the merged ISIC test set (400 images). Each component is removed in turn from the complete architecture; Δ columns are reported against the full model in percentage points, and ΔHD95 as an absolute difference. The best result in each column is in **bold**.

Configuration	Dice	ΔDice	IoU	Boundary IoU	ΔBIoU	HD95
Full (LCE + DCT + Boundary)	**0.9082**	–	**0.8451**	**0.4716**	–	**11.27**
w/o DCT	0.8996	−0.86	0.8338	0.4616	−0.99	12.22
w/o Boundary	0.9016	−0.66	0.8359	0.4430	−2.85	11.68
w/o LCE	0.9020	−0.61	0.8359	0.4648	−0.67	12.33
w/o diagonal scans	0.9056	−0.25	0.8407	0.4560	−1.56	11.35

**Table 5:** Cross-domain generalization on a held-out 200-image HAM10000 test subset. All six models are trained only on the merged ISIC training set and evaluated with four-flip test-time augmentation; no domain-specific fine-tuning is performed. The best result in each column is in **bold**.

Model	Dice	IoU	Acc	Sen	Spe	HD95
U-Net (ResNet-34) [5]	0.9333	0.8799	0.9711	0.9340	0.9819	7.95
Att-UNet [6]	0.9310	0.8775	0.9710	0.9268	0.9839	8.15
VM-UNet [14]	0.8638	0.7950	0.9440	0.8231	0.9793	19.30
TransUNet [9]	0.9172	0.8588	0.9645	0.8920	**0.9856**	10.05
MALUNet [27]	0.8645	0.7814	0.9486	0.8886	0.9660	16.24
**Hydra-DermSeg-Net (ours)**	**0.9347**	**0.8824**	**0.9720**	**0.9344**	0.9830	**7.77**

## Data Availability

This study used only publicly available datasets. The ISIC 2017 and ISIC 2018 challenge datasets can be accessed through the International Skin Imaging Collaboration archive (https://challenge.isic-archive.com/ accessed on 22 July 2026), and the HAM10000 dataset is available from the Harvard Dataverse (https://doi.org/10.7910/DVN/DBW86T accessed on 22 July 2026). No new data were created in this study. The source code, the split files, the de-duplication lists and the trained model weights are available from the corresponding author upon reasonable request.

## Abbreviations

The following abbreviations are used in this manuscript:

CNN	Convolutional Neural Network
SSM	State-Space Model
DCT	Discrete Cosine Transform
CLAHE	Contrast-Limited Adaptive Histogram Equalization
LCE	Learnable Contrast Enhancement
ISIC	International Skin Imaging Collaboration
HAM10000	Human Against Machine with 10000 training images
IoU	Intersection-over-Union
HD95	95th-Percentile Hausdorff Distance
SMP	Segmentation Models PyTorch
